# Understanding the underuse: Barriers to vacuum-assisted birth among clinicians in Tanzania: An exploratory qualitative study

**DOI:** 10.1371/journal.pone.0354738

**Published:** 2026-07-27

**Authors:** Lydia S. Massae, Rashidi Heri Kiangi, Victor Zeno Chikwala, Ally Abdul Lyimo, Stella Emmanuel Mushy, Lilian Teddy Mselle

**Affiliations:** 1 Department of Midwifery, Child and Reproductive Health, Muhimbili University of Health and Allied Sciences, Dar es Salaam, Tanzania; 2 Department of Nursing Education and Management, Muhimbili University of Health and Allied Sciences, Dar es Salaam, Tanzania; Jhpiego, UNITED STATES OF AMERICA

## Abstract

**Background:**

Vacuum-assisted birth (VAB) is a critical obstetric intervention for saving the lives of the mothers and newborns, particularly in cases of prolonged second-stage labor, maternal exhaustion, or fetal distress. Despite its proven benefits, VAB remains underutilized, contributing to an increased rate of unnecessary cesarean sections, some of which could be avoided with timely vacuum extraction. This study aimed to explore the barriers to the use of VAB among clinicians at a tertiary hospital in Tanzania.

**Materials and Methods:**

An exploratory qualitative study using in-depth interviews (IDIs) was employed. Twelve clinicians with relevant experience in vacuum-assisted birth were recruited purposively into the study. A semi-structured IDI guide was used to facilitate the interviews. Data were analyzed inductively using thematic analysis to identify key patterns and categories related to barriers in the use of VAB.

**Results:**

Organizational and individual barriers were identified as key challenges to the use of VAB. Organizational barriers included insufficient availability of VAB equipment, ineffectiveness of the available devices, and poor team dynamics that hindered collaborative decision-making regarding VAB use. Individual barriers centered on clinicians’ apprehension about potential adverse outcomes, along with limited skills and practical experience in performing VAB procedures.

**Conclusion:**

The utilization of VAB by clinicians is hindered by both organizational and individual barriers. Addressing these challenges through improved availability of functional equipment, enhanced training to build clinicians’ skills, and improving working conditions has the potential to increase the use of VAB. Strengthening these areas could help reduce the rate of unnecessary caesarean deliveries.

## Introduction

Vacuum-assisted birth (VAB) plays a critical role in improving maternal and newborn outcomes, particularly during complicated deliveries, by reducing the need for cesarean sections (CS). The procedure involves applying a suction device to the infant’s head to facilitate its passage through the birth canal [1,2]. VAB is especially important in settings with limited access to surgical interventions, offering a safe and effective alternative to CS when performed by skilled healthcare providers [1,3]. Clinical indications for VAB include prolonged second stage of labor, maternal exhaustion, or signs of fetal distress [4]. When used appropriately, it can mitigate the risks associated with unnecessary cesarean deliveries, especially in low-resource settings where CS-related complications are harder to manage [4,5].

Recent data indicate a considerable rise in the prevalence of caesarean sections (CS) in Tanzania over the past two decades. The 2022 Tanzania Demographic and Health Survey (TDHS) reported a nationwide cesarean section prevalence of 10.4%, up from about 4% in 2004/05, indicating a significant upward trend in surgical deliveries [6]. A 2024 systematic review and meta-analysis of 26 studies, encompassing over 600,000 women in Eastern Africa, reported a pooled regional prevalence of cesarean sections (CS) of 24.0%, with significant variability across nations and healthcare facility tiers [7].

Although caesarean section rates have steadily increased in Tanzania, VAB remains rarely performed. In 2020, at Muhimbili National Hospital, vaginal assisted births (VAB) constituted merely 0.8% of deliveries, whereas 54% were performed via cesarean section, highlighting a significant disparity in the utilization of operative birth procedures [8]. Evidence from implementation studies demonstrates that this low utilization is not inevitable.

Studies indicate that up to 52% of clinicians lack sufficient knowledge and confidence to perform VAB, which significantly hinders its integration into routine obstetric care [9–12]. However, following provider training and equipment support in 15 health facilities in Tanzania, VAB increased from almost zero to 2%, while caesarean section rates remained stable at 10–11%, suggesting that improved capacity can increase the appropriate use of VAB [13]. These findings highlight an important gap between the increasing use of caesarean section and persistently low use of VAB in Tanzania, underscoring the need to understand clinician-level barriers to VAB utilization.

Despite its benefits, evidence of complications associated with VAB may contribute to its limited use by many clinicians. Both maternal and neonatal risks are documented, including cephalhematoma, scalp lacerations, and postpartum haemorrhage [14,15]. These potential complications may influence clinicians to avoid VAB in favor of other delivery methods.

Tanzania has included VAB in its national maternal, reproductive, and child health guidelines as a recommended intervention for skilled healthcare providers [3,16–18]. Nevertheless, VAB use continues to decline despite the availability of clear guidelines and supportive evidence [19–21]. This underutilization may be contributing to the increasing rates of CS deliveries in the country.

There is limited data on the specific barriers to VAB use at the tertiary facility level in Tanzania. Understanding these barriers is essential to designing targeted interventions that could promote the safe and appropriate use of VAB. Therefore, this study aimed to gain an in-depth understanding of why clinicians opt not to use VAB even when it is clinically indicated and instead prefer CS.

## Materials and methods

### Study design and setting

We conducted an exploratory qualitative study to analyze the barriers to VAB use among clinicians at Tanzania’s tertiary maternity hospital. The hospital manages approximately 9,000 deliveries annually and serves as a referral center, with 60% of patients arriving from other facilities. Notably, 75% of all deliveries are performed through emergency CS [22]. Despite the hospital’s high delivery volume and capacity for specialized obstetric care, data from the District Health Information System (DHIS2) show consistently low VAB utilization: 2.0% in 2020, 2.2% in 2021, and 2.1% in 2022. The exploratory design of this study enabled an in-depth understanding of clinicians’ experiences and the contextual factors influencing the limited use of VAB in this setting. During the study period, VAB at the study site was performed using reusable rigid stainless steel vacuum cups connected via tubing to an external hand-operated vacuum pump that generated the negative pressure required for assisted vaginal birth. The reusable metal cups were sterilized between uses in accordance with the hospital’s infection prevention and control protocols.

### Participants and recruitment

We purposively recruited 12 clinicians, comprising obstetricians and residents working in the labor ward [23], with assistance from the ward in charge. Obstetricians who participated in the study were required to possess a minimum of six months of experience in the labour ward following their specialist training. Residents enlisted in the study were those who had completed a minimum of one year of residency training and were acquiring specialist training in obstetrics. The ward in charge initially identified potential participants, after which the researcher approached them in person during or after their clinical shifts. The purpose and procedures of the study were clearly explained, and those who expressed interest were provided with review details and invited to schedule sessions at their convenient times and locations. All approached participants agreed to participate in the study. Participants who consented to take part were given a written informed consent form, which they signed to verify their participation. Recruitment continued until data saturation was achieved [24], which occurred after 10 interviews, with the final 2 confirming no new insights were generated.

### Data collection tools and procedures

A semi-structured interview guide was developed and used as the primary data collection tool to understand barriers to VAB use among clinicians. The interview guide was created following a structured literature review that analyzed obstacles to VAB utilization. Qualitative and mixed-methods research were examined to discover recurring domains affecting VAB use. The domains discovered from the literature were aligned with the socioecological model, which informed the organization of the interview guide. At the individual level, the inquiry examined doctors’ knowledge, skills, training exposure, confidence in executing VAB, perceived competence, and risk perception. At the organizational level, inquiries focused on the availability of functional equipment, guidelines and protocols, workload, staffing levels, and the facility culture about operative vaginal birth. The study involved native Kiswahili speakers, necessitating the translation of a tool initially developed in English to ensure comprehension and clarity in the local clinical context. Bilingual research team members, skilled in obstetrics, rigorously reviewed the translated tool, focusing on terminology related to vacuum-assisted birth and hospital procedures to maintain consistency with the language used in Tanzanian hospitals. When direct translations were absent, adjustments were made to use locally understood expressions. The Kiswahili version was validated by clinicians similar to study participants, with feedback emphasizing clarity, cultural relevance, and vocabulary appropriateness. Minor modifications were made to align questions with local clinical practices and communication styles.

The data collection process was conducted from March 10 to May 24, 2023. In-depth interviews took place in a private setting within the hospital, chosen by each participant to ensure confidentiality and minimize interruptions. Each interview lasted between 40 and 50 minutes.

A trained research assistant (RA), holding a professional background in nursing and midwifery, fluent in Kiswahili, and experienced in health-related research, supported the data collection. Before data collection, the RA participated in a one-day training session that covered study objectives, ethical research practices, effective interviewing techniques, and the use of the interview guide. Moreover, the researcher (LSM) spearheaded data collection procedures, including obtaining consent from participants, setting appointments, and conducting interviews.

During the interviews, both the principal investigator and the RA employed active listening skills and neutral probing skills to encourage participants to elaborate on their responses. All interviews were audio-recorded with participants’ consent and supplemented with field notes to capture non-verbal cues and contextual details. This rigorous data collection process ensured the credibility and depth of qualitative data gathered for the study.

### Data analysis

The interviews were audio-recorded and transcribed verbatim. We employed thematic analysis guided by the socio-ecological model (SEM) [25,26]. We conducted the analysis manually in Microsoft Excel for systematic data management. The multidisciplinary research team performed iterative coding, with senior researchers verifying the accuracy of codes [27]. To provide early insights and guide the focus of subsequent in-depth interviews, data collection and analysis were conducted simultaneously. This approach ensured that the identified themes were closely linked to the data and revealed barriers to the use of VAB, as generated from the interviews. Transcripts were read repeatedly to properly understand the information on VAB use. The researchers coded one transcript, generated codes that were discussed and agreed upon, and then developed the codebook. Researchers coded independently by using the codebook and letter discussed to agree on the generated codes. Agreed codes were examined and grouped based on their similarities and differences to form subthemes and then themes regarding barriers to performing VAB. The analysis used Kiswahili transcripts to maintain participants’ meaning, with translation to English occurring only during reporting.

### Ethical considerations

Ethical clearance for this study was obtained from the Muhimbili University of Health and Allied Sciences Institutional Review Board with Ref. No. DA.282/298/01.C/2065. Permission for data collection was obtained from the Clinical Research, Training, and Consultancy Unit at MNH with a ref. No. MNH/CRTCU/Perm/2023/119. Written informed consent was obtained from the participants before commencing the interview. Participants were informed of the benefits, possible risks, procedures, and voluntary participation in the study. Permission for audio recording was obtained from the participants before conducting the interview. To ensure confidentiality, the interviews were conducted in a comfortable place identified by the participants themselves. All participants were assigned unique identifiers for concealment of identity.

## Results

### Demographic characteristics of the study participants

A total of twelve participants were purposefully included in the study, comprising medical specialists and seven resident doctors working in the labor ward. The participants’ professional experience ranged from six months to over thirty years. A summary of participants’ demographic and professional characteristics is presented in S1 Table.

Thematic analysis yielded two overarching themes: organizational and individual barriers to the utilization of VAB among clinicians. These themes, along with their corresponding subthemes, are outlined in S2 Table and elaborated in the subsequent sections.

### Organizational barriers

This theme encompasses the systemic obstacles within the healthcare facility that impede the effective implementation and adoption of VAB. Three subthemes emerged under this category: malfunctioning and inaccessible medical equipment, team dynamics in deciding when to use VAB, and limited accessibility and utilization of VAB guidelines and SOP.

### Malfunctioning and inaccessible medical equipment

Participants reported that during the second stage of labor, when indications for VAB use were present, necessary equipment was often unavailable or missing at the critical moment. Moreover, the limited supply of available equipment was cited as a contributing factor to the underutilization of VAB within the ward. Reflecting on this issue, one interviewee said;

“*Here in the labor ward, we don’t have many tools. However, I believe that we would benefit from having much of this equipment available to us if we approach these situations positively. As it currently stands, we only have one tool.” (Resident, 6 years of experience IDI 2*)

Participants also pointed out that not regularly checking the functional equipment in the ward is a major barrier to the effective use of VAB. This led to the presence of defective and inappropriate equipment, which later malfunctioned at a critical moment. These were narrated as follows.

*“The availability of equipment has been a persistent challenge in [hospital], especially in the labor ward. The vacuum cups often fail to grasp the baby’s head properly, and the pumps are damaged. However, the process of locating and replacing this equipment is ineffective. Sometimes, the items are simply unavailable in the ward, and replacement depends on the hospital’s procurement system, which can be slow and unreliable.” (Specialist, 18 years of experience, IDI 8)*

*“…Additionally, because of the poor condition of our equipment, the cup tends to fall out when we take out the baby.” (Specialist, 21 years of experience, IDI 6)*

Clinicians also revealed that the emergency tray lacked the complete set of VAB equipment necessary for the procedure. This unavailability was attributed to improper assembly and storage of the equipment, which in turn discouraged the use of VAB. One participant stated that;

*“… For instance, when you are using a vacuum to deliver a baby and need to change the cup, you discover that it is too small for the baby’s head and that it may take a while to find a larger one and might never find the proper size”* (*Resident, 9 years of experience* IDI 3)

Unfamiliarity with the type of VAB equipment was mentioned to be another barrier to the utilization of VAB. This was a concern when the clinician was not used to the VAB equipment, which is available in the ward. The participant said;

*"I can say that these vacuum devices vary quite a bit, so sometimes you may come across a device that you’re not used to using for assisting with delivery. At times, you find that the cup is too small. Therefore, I would suggest that they provide a wider range of sizes to enable us to perform the procedure more effectively*."(*Resident, 12 years of experience* IDI 10)

### Team dynamics in deciding when to use VAB

Clinicians reported that the existence of different viewpoints from members of the team, the minimal consensus in team decisions, and delays in decision-making prohibit VAB use. Several participants noted that differing viewpoints within the team regarding the use of VAB during the second stage of labor were largely driven by fears of negative outcomes, such as cephalhematoma and low Apgar score, for which clinicians might be held accountable. Talking about this issue, an interviewee said;

“*There are conflicting ideas among ourselves; for example, you may decide to help the client by using the vacuum, but another person may say, “No, let’s not help her.” Fearing that the child’s outcome will be poor and that you will be held responsible, you may be asked why you did not send her for a caesarian section sooner…” (Specialist, 18 years of experience, IDI 8)*

Another participant added that;

“*There may be a few differences between the doctor and the nurse while exchanging ideas and their views because each has unique experiences and knowledge, but the doctor makes the final decision” (Resident, 9 years of experience, IDI 11)*

Participants revealed that within the team, there is an existence of minimal consensus decisions in performing VAB. It was evident that this was because of low practice among clinicians and the absence of clear, standardized protocols within the labor ward. One participant commented that:

*“Several SOPs have been posted, such as one for eclampsia. If we post for vacuum delivery, we will all be aware very well that when I see this mother needs this service one hundred percent, and our decision becomes uniform, we know what to do better than waiting for another person to come and make decisions, which in turn delays the provision of services” (Resident, 6 years of experience, IDI 2)*

Some participants raised concerns about delays in decision-making by clinicians, especially the residents. Participants have shown to be reluctant to act quickly when the need arises from a fear of making the wrong call due to a lack of approval from supervisors and more experienced clinicians. An experienced resident with 6 years of experience shared the following:

“…*There may be a vacuum case, but they tend to call in special persons so and so to attend to the client; as a result, you realize that the same individuals are managing the issue over and over, leaving others to be observers rather than take action.” (Resident, 6 years of experience, IDI 2)*

### Limited accessibility and utilization of VAB guidelines and SOPs

This subtheme highlights systemic challenges related to the limited availability, visibility, and use of clinical guidelines and standard operating procedures for VAB. Participants reported that existing guidelines were not consistently accessible within the ward, making it difficult for clinicians, especially those with less clinical experience, to refer to them during clinical decision-making. Several participants noted that the available VAB guidelines appeared to be designed with more experienced practitioners in mind, often lacking practical clarity for those still building competency. One participant said

*“It is simple for you to grasp the guidelines if you are an expert in the field and read them because of your knowledge. However, other people you may come across may not have had the opportunity to access certain resources.” (Resident, 9 years of experience, IDI 11).*

In addition, an expert obstetrician echoed.

*“There are guidelines, but very few people adhere to them, especially those who are capable of this vacuum delivery, because many clients would otherwise have to undergo caesarian sections.” (Specialist, 15 years of experience, IDI 5)*

This perceived complexity may discourage use among junior clinicians, reducing adherence and undermining confidence in guideline-based practice.

In addition to content challenges, participants also emphasized the physical inaccessibility of the guidelines. SoPs and protocols were often not displayed in delivery areas or easily retrievable when needed. This absence of visual reinforcement and practical guidance in the clinical environment further weakened their role in routine practice. Participants explained:

*“…as far as I can tell, there are no posted instructions or standard operating procedures (SOPs) in the ward on when and how to perform a vacuum delivery.” (Specialist, 15 years of experience, IDI 5)*

Moreover, there appeared to be a broader organizational gap in the promotion and reinforcement of VAB SOPs. Clinicians reported minimal emphasis on the importance of following protocols, both in supervision and ongoing training. This lack of institutional encouragement contributed to inadequate use and a general undervaluing of guidelines in day-to-day obstetric care. Emphasizing the importance of displaying SoPs. The participant stated:

*“The existing SOPs should be posted; they are not too many. Right now, they’re just on paper, but if we display them, even someone who has forgotten can refer to them—just like we display guidelines for eclampsia and other conditions. So, in cases where someone needs assistance during delivery, it should show where to place the cup, how many times to pull, and so on. These should also be displayed in the maternity ward.” (Specialist, 16 years of experience, IDI 9)*

Overall, the limited accessibility and utilization of clinical guidelines reflected a broader organizational shortfall, hindering the standardization and safe implementation of VAB across clinical teams.

### Individual barriers

Under this theme, apprehension regarding adverse outcomes and inadequate skills and experience in performing VAB resulted in the nonuse of VAB among clinicians in the second stage of labor. The subthemes are described below.

### Apprehension regarding adverse outcomes following VAB

This subtheme reflects clinicians’ diminished confidence and lack of enthusiasm to participate in VAB, frequently influenced by prior poor experiences and apprehension regarding adverse outcomes. Participants articulated ongoing apprehensions about the potential hazards linked to the procedure, especially about the welfare of both the mother and the infant. This fear often led to reluctance to commence VAB, despite clinical indications for its necessity. As one specialist explained:

*“…[Clinicians] are hesitant to use VAB because, if used incorrectly, it could tear the woman’s cervix, causing severe bleeding (PPH) and other serious medical conditions...” (Specialist, 15 years of experience, IDI 5)*

Participants noted that anxiety concerning the client’s condition has contributed to the development of a team culture that discourages the use of VAB even when clinically indicated. This reluctance stems from fears that attempting VAB might lead to complications or delays in delivering the baby. This concern is illustrated in the following quote.

*“…Even when it’s necessary, our team rarely utilizes the vacuum too frequently, and I believe this is because we’re scared of the potential consequences for the mother and the child…” (Resident, 8 years of experience, IDI 4)*

Participants expressed fear of the negative outcomes related to VAB use, which was attributed to a prevailing culture of blame within the clinical environment. Clinicians reported that this culture discourages the use of VAB, as individuals fear being held personally accountable in the event of an adverse outcome. As a result, the procedure is rarely preferred or utilized in the ward. A participant stated that;

*“…When using a vacuum, there’s a risk of being held accountable. This blame culture has made doctors reluctant to utilize vacuums or engage in any related practices since they are unaware of them, which makes them dislike performing VABs as time passes.” (Resident, 10 years in experience, IDI 12)*

It was pointed out that many clinicians tend to avoid using VAB due to the belief that the equipment is unreliable. The anticipation that the vacuum extractor might fail to function effectively contributes to a lack of confidence in the procedure, ultimately discouraging clinicians from considering VAB as a viable option during delivery. A participant said;

*“To be honest, doctors feel that this method will not work and hence prefer not to use a vacuum in assisting the mother since their experience in frequently fails; as a result, rather than performing the procedure, you will find that they immediately prepare the patient for surgery” (Resident, 12 years in experience, IDI 10)*

Participants reported that the requirement to document and justify negative outcomes following failed VAB procedures acts as a deterrent to its use. The fear of having to provide detailed explanations in the event of an unsuccessful attempt often leads clinicians to opt for alternative modes of delivery. As one resident stated:

*“Any adverse outcome must be documented in a report explaining why the mother died, why the baby died, or why the baby had a low Apgar score. Consequently, clinicians fear being questioned or blamed later for why the baby did not thrive or why the delivery had a poor outcome. This fear of blame is particularly strong among private patients, who constitute the majority of cases here. This culture of blame has led to widespread reluctance to perform vacuum-assisted births.” (Residents, 8 years’ experience, IDI 1)*

### Insufficient skills and experience in performing VAB

Many participants indicated that limited clinical experience among junior professionals, few numbers of clinicians proficient in VAB, and the infrequency of VAB-indicated cases in the labor ward collectively contribute to its underutilization. Inadequate preparation and doubts about performing the procedure further discourage its use. A participant said:

*“I feel the primary issue might be decision-making—whether to use a vacuum or not. Beyond the decision itself, it also involves the clinician’s knowledge and confidence in performing the procedure. You find that someone knows they should perform it, but they are not comfortable doing so, possibly because they are not fully familiar with the technique or they fear the potential outcomes” (Specialist, 16 years of experience, IDI 9)*

Participants emphasized that VAB requires specific technical competencies, which are typically acquired through hands-on experience and mentorship. These skills are more commonly found among experienced clinicians who are familiar with established protocols and adhere to best practices. As a result, clinicians who lack adequate training, clinical experience, and exposure to the procedure are more likely to underutilize VAB. This was echoed by a participant who said;

*“…our experience is limited in the first place because of the limited time we spend in the labor ward and the few period sessions we get while at university” (Resident, 6 years’ experience, IDI 12)*

Participants indicated that only a limited number of personnel in the labor ward have sufficient competence with VAB. It was observed that most clients are overseen by specialists and midwives with extensive experience in the ward, resulting in more proficiency in the procedure. Conversely, some clinicians, especially junior personnel, have restricted practical opportunities to perform VAB, which impedes their capacity to develop proficiency and assurance in its application. This was narrated as follows.

*“I can say that there are two main reasons why a clinician might not be able to use a vacuum: either they have never used it before or they lack the confidence to use it, which could result in them being unable to use it even if all the indicators are present” (Specialist, 21 years’ experience, IDI 7)*

Participants reported that the occurrence of a few cases in the labor that need VAB has resulted in low VAB usage. Most cases that arrive at the hospital tend to end in emergency procedures, including the caesarean section.

*“…although there aren’t many cases, I believe that we should provide time for practice by utilizing manikins; we might schedule time, perhaps once a month, to gather clinicians, go to simulation rooms, and demonstrate with Mankins, and by doing this periodically, we can develop our skills and improve as a team” (Resident, 8 years of experience, IDI 4).*

Participants noted that individual motivation to engage in VAB practice was generally low, often requiring clinicians to invest additional personal time. Furthermore, the short duration allocated to ward rounds and educational sessions was identified as a contributing factor to the low frequency of VAB performance. This was narrated as follows:

*“What is required is a personal effort from the clinicians themselves and must be able to set aside extra time to read through the guidelines and find time to come into labor ward to learn what is going on and acquire more experience” (Resident, 12 years experience, IDI 10)*

*“… as doctors, we have many duties in the ward and elsewhere, and we have very little time in labor. As a result, you don’t have a lot of opportunity to practice in real life because there isn’t much equipment available.” (Specialist, 21 years of experience, IDI 6)*

These findings collectively demonstrate how past negative experiences, whether personally experienced or institutionally recorded, cultivate a cycle of doubt and disengagement, obstructing the development of confidence and motivation essential for effective VAB practice.

## Discussion

This study highlighted both organizational and individual barriers that prevent clinicians from effectively using VAB, an important procedure for managing certain cases of the prolonged second stage of labor. These challenges not only delay timely and appropriate care but also lead to an increased number of CS. The findings point to critical areas within the healthcare system that require focused attention and improvement to support safer and more effective childbirth practices.

### Organizational-related factors affecting VAB use

Clinicians frequently reported that malfunctioning equipment, lack of regular maintenance, and the general unavailability of VAB devices significantly hindered their ability to offer this critical intervention. In some instances, inappropriate sizes of vacuum extractor cups have resulted in slippage during procedures, undermining clinicians’ confidence in the use of VAB. These constraints often required a shift to CS, even though a vaginal-assisted birth would have been safer and more appropriate.

Such equipment-related constraints may contribute to increased risks of fetal injury, maternal infections, postpartum hemorrhage, and prolonged hospitalization for both mother and infant due to delayed or inappropriate delivery methods. These findings point to the urgent need for routine equipment inspections, preventive maintenance protocols, and policies that guarantee the availability of functioning vacuum devices in all delivery rooms.

These results are consistent with reports from Latin America, sub-Saharan Africa, and Asia, where a lack of functional equipment has been linked to underuse of VAB and increased rates of CS [21]. Similar patterns have been observed in Tanzania, where residents expressed low confidence in using vacuum extractors, largely due to frequent equipment failures and inadequate maintenance [28,29]. In addition, defective devices were reported to negatively influence healthcare providers’ expectations and hands-on experience with vacuum delivery [20,22].

### Individual-related barriers affecting VAB use

A notable finding from this study was the influence of team dynamics on decision-making regarding VAB in the labor ward. Hierarchical structures often left junior staff feeling disempowered or hesitant to suggest or advocate for VAB, leading to missed opportunities for timely interventions. Effective VAB use requires collaborative decision-making to ensure safe and timely delivery. In the absence of a clear consensus among team members, critical decisions may be delayed, prolonging the second stage of labor and increasing the risk of fetal compromise.

To improve this, clinicians emphasized the importance of fostering professional collaboration, developing clear clinical decision-making protocols, and empowering all qualified team members, regardless of rank, to recommend or initiate VAB when clinically appropriate. Participants also stressed the need for accessible standard operating procedures (SOPs), regular practice alongside experienced colleagues, and unambiguous communication to reduce uncertainty and delays. These findings align with those documented in Italy [30], which noted that even experienced specialists often rely on intuitive decision-making rather than formal guidelines, especially in time-sensitive clinical scenarios.

Clinicians also expressed deep concerns about potential complications associated with VAB, Such as neonatal trauma or failed procedures. These fears were often reinforced by a workplace culture that assigns blame in the event of adverse outcomes, further discouraging the use of VAB. This apprehension contributes to a tendency toward excessive caution, resulting in increased reliance on CS even when VAB would be a suitable option. Similar concerns have been reported by other researchers in Tanzania and South Africa, where fear of failure and professional repercussions associated with unsuccessful VAB attempts have negatively impacted clinicians’ confidence and career progression [20,22]. Such hesitations can delay necessary interventions and increase maternal morbidity due to avoidable surgical deliveries. To address these issues, the study suggests a shift toward non-punitive clinical governance that includes supportive supervision, regular case reviews, and debriefing sessions. These practices can help staff reflect on adverse events constructively, promote shared learning, and build confidence in using VAB without fear of blame.

Inadequate skills and experience were predominantly observed among younger professionals. The execution of VAB during the second stage of labor requires proficiency, confidence, and clinical experience to ensure a favorable outcome. Clinicians reported limited opportunities to practice VAB in the ward due to the infrequent occurrence of eligible cases, which resulted in insufficient hands-on experience. This challenge is further compounded by inadequate training for clinicians in the labor ward and a lack of structured monitoring of VAB use.

Another issue identified was the absence of experienced clinicians available to attend to clients requiring VAB and to provide supervision or conduct assessments when needed. Studies from Tanzania and findings from a systematic review support these observations, revealing that many young professionals lack the confidence, competence, and expertise needed to perform VAB, often due to previous adverse outcomes associated with the procedure [21,22,29]. These factors contribute to uncertainty and delays in decision-making, potentially compromising the safety of both the mother and the infant. Addressing these gaps requires individual commitment to ongoing practices, as well as a strong understanding of established protocols and clinical guidelines for safe and effective VAB.

Overall, the findings underscore that the safe use of VAB depends on accurate case selection and assessment, strict adherence to standard procedures, proficiency in equipment handling, and consistent application of clinical expertise. These elements should be emphasized in routine ward practices and incorporated into continuous professional development initiatives.

### Study strengths and limitations

This study provides valuable insights into the key challenges that make it difficult to use VAB. By using a qualitative approach, we were able to hear directly from health workers about their real-life experiences with the limited use of VAB in clinical settings, which made the findings more meaningful and grounded

However, the study had several limitations. First, it involved a small number of participants from selected health facilities, which may limit the generalizability of the findings to other regions or health systems. To address this, participants were purposefully selected to represent diverse professional roles and levels of experience to enhance the richness of the data. Second, social desirability bias may have influenced responses, as participants might have shared views they believed were expected. To mitigate this, interviews were conducted in a private setting, and confidentiality was assured to encourage honest responses. Finally, the qualitative design captures in-depth perceptions but does not provide measurable outcomes. Future studies could use surveys or other tools to measure how common these challenges are in larger populations.

## Conclusion

Both organizational and personal-related challenges made it difficult for health workers to use VAB. Health centers lacked proper equipment or had nonfunctional tools, and weak teamwork affected decision-making. On an individual level, some health workers were worried about possible complications, while others lacked the training and experience needed to feel confident using the procedure. Addressing these issues is essential to improve the safe and effective use of this important treatment.

## Supporting information

S1 Table Demographic and professional characteristics of participants. (TIF)

S2 Table Themes and subthemes on barriers to the use of VAB. (TIF)

S3 Text Excerpts. (PDF)

## References

[1] Tonismae T, Canela CDGW. Procedure for vacuum-assisted vaginal birth. StatPearls. Treasure Island (FL): StatPearls Publishing. 2023:1.

[2] Crossland N, Kingdon C, Balaam M-C, Betrán AP, Downe S. Women’s, partners’ and healthcare providers’ views and experiences of assisted vaginal birth: a systematic mixed methods review. Reprod Health. 2020;17(1):83. doi: 10.1186/s12978-020-00915-w 32487226 PMC7268509

[3] Nolens B, Capelle M, van Roosmalen J, Mola G, Byamugisha J, Lule J, et al. Use of assisted vaginal birth to reduce unnecessary caesarean sections and improve maternal and perinatal outcomes. Lancet Glob Health. 2019;7(4):e408–9. doi: 10.1016/S2214-109X(19)30043-9 30879501

[4] Deutchman M. Vacuum extraction: a necessary skill. American family physician. 2000;1269–1270,1273-1274,1276. 11011857

[5] SS M. Vacuum Assisted Vaginal Delivery: Incidence, Maternal and Neonatal Complications in Muhimbili National Hospital, Dar Es Salaam, Tanzania. Muhimbili University. 2016.

[6] Nahayo B, Olorunfemi G, Ndayishimye S, Nsanzabera C. Prevalence and factors associated with caesarean section among Tanzanian women of reproductive age: evidence from the 2022 Tanzania demographic and health survey data. BMC Public Health. 2025;25(1). doi: 10.1186/s12889-025-21967-2 40016698 PMC11866573

[7] Habteyes AT, Mekuria MD, Negeri HA, Kassa RT, Deribe LK, Sendo EG. Prevalence and associated factors of caesarean section among mothers who gave birth across Eastern Africa countries: Systematic review and meta-analysis study. Heliyon. 2024;10(12):e32511. doi: 10.1016/j.heliyon.2024.e32511 38952380 PMC11215273

[8] Makokha-Sandell H, Mgaya A, Belachew J, Litorp H, Hussein K, Essén B. Low use of vacuum extraction: Health care Professionals’ Perspective in a University Hospital, Dar es Salaam. Sex Reprod Healthc. 2020;25:100533. doi: 10.1016/j.srhc.2020.100533 32505920

[9] Nolens B, Lule J, Namiiro F, van Roosmalen J, Byamugisha J. Audit of a program to increase the use of vacuum extraction in Mulago Hospital, Uganda. BMC Pregnancy Childbirth. 2016;16(1):258. doi: 10.1186/s12884-016-1052-3 27590680 PMC5010743

[10] Chang X, Chedraui P, Ross MG, Hidalgo L, Peñafiel J. Vacuum assisted delivery in Ecuador for prolonged second stage of labor: maternal-neonatal outcome. J Matern Fetal Neonatal Med. 2007;20(5):381–4. doi: 10.1080/14767050701227927 17674241

[11] Ayala-Yáñez R, Bayona-Soriano P, Hernández-Jimenez A, Contreras-Rendón A, Chabat-Manzanera P, Nevarez-Bernal R. Forceps, actual use, and potential cesarean section prevention: study in a selected Mexican population. J Pregnancy. 2015;2015:489267. doi: 10.1155/2015/489267 26380111 PMC4561325

[12] Geelhoed D, de Deus V, Sitoe M, Matsinhe O, Lampião Cardoso MI, Manjate CV, et al. Improving emergency obstetric care and reversing the underutilisation of vacuum extraction: a qualitative study of implementation in Tete Province, Mozambique. BMC Pregnancy Childbirth. 2018;18(1):266. doi: 10.1186/s12884-018-1901-3 29945551 PMC6020342

[13] Dominico S, Bailey PE, Mwakatundu N, Kasanga M, van Roosmalen J. Reintroducing vacuum extraction in primary health care facilities: a case study from Tanzania. BMC Pregnancy Childbirth. 2018;18(1):248. doi: 10.1186/s12884-018-1888-9 29914412 PMC6006733

[14] Chawanpaiboon saifoncha S, Chawanpaiboon S, Titapant V, Pooliam J. Maternal complications and risk factors associated with assisted vaginal delivery. BMC Pregn Childbirth. 2023;9(9):1–9. doi: 10.1186/s12884-023-05999-9 PMC10601252 37884886

[15] Chawanpaiboon S, Titapant V, Pooliam J. Neonatal complications and risk factors associated with assisted vaginal delivery. Sci Rep. 2024;14(1):11960. doi: 10.1038/s41598-024-62703-x 38796580 PMC11127920

[16] MoHCDGEC. National plan for reproductive, maternal, newborn, child and adolescent health & nutrition (2021/2022 - 2025/2026) One plan III. 2022.

[17] Eze P, Lawani LO, Chikezie RU, Ukaegbe CI, Iyoke CA. Perinatal outcomes of babies delivered by second-stage Caesarean section versus vacuum extraction in a resource-poor setting, Nigeria - a retrospective analysis. BMC Pregnancy Childbirth. 2020;20(1):298. doi: 10.1186/s12884-020-02995-9 32410592 PMC7227301

[18] Dominico S, Kasanga M, Mwakatundu N, Chaote P, Lobis S, Bailey PE. Factors related to the practice of vacuum-assisted birth: findings from provider interviews in Kigoma, Tanzania. BMC Pregnancy Childbirth. 2021;21(1):302. doi: 10.1186/s12884-021-03738-0 33853540 PMC8048302

[19] Dominico S, Bailey PE, Mwakatundu N, Kasanga M, van Roosmalen J. Reintroducing vacuum extraction in primary health care facilities: a case study from Tanzania. BMC Pregnancy Childbirth. 2018;18(1):248. doi: 10.1186/s12884-018-1888-9 29914412 PMC6006733

[20] Feeley C, Crossland N, Betran AP, Weeks A, Downe S, Kingdon C. Training and expertise in undertaking assisted vaginal delivery (AVD): a mixed methods systematic review of practitioners views and experiences. Reprod Health. 2021;18(1):92. doi: 10.1186/s12978-021-01146-3 33952309 PMC8097768

[21] Bailey PE, van Roosmalen J, Mola G, Evans C, de Bernis L, Dao B. Assisted vaginal delivery in low and middle income countries: an overview. BJOG. 2017;124(9):1335–44. doi: 10.1111/1471-0528.14477 28139878

[22] Makokha-Sandell H, Mgaya A, Belachew J, Litorp H, Hussein K, Essén B. Low use of vacuum extraction: Health care Professionals’ Perspective in a University Hospital, Dar es Salaam. Sex Reprod Healthc. 2020;25:100533. doi: 10.1016/j.srhc.2020.100533 32505920

[23] Francis JJ, Johnston M, Robertson C, Glidewell L, Entwistle V, Eccles MP, et al. What is an adequate sample size? Operationalising data saturation for theory-based interview studies. Psychol Health. 2010;25(10):1229–45. doi: 10.1080/08870440903194015 20204937

[24] Creswell JW. Qualitative Inquiry Research Design. 3rd ed. Lauren Habib, Kalie Koscielak, Brittany Bauhaus MM, editor. London: SAGE Publications, Inc; 2017:1–472

[25] Moser A, Korstjens I. Series: Practical guidance to qualitative research. Part 3: Sampling, data collection and analysis. Eur J Gen Pract. 2018;24(1):9–18. doi: 10.1080/13814788.2017.1375091 29199486 PMC5774281

[26] Nowell LS, Norris JM, White DE, Moules NJ. Thematic analysis: striving to meet the trustworthiness criteria. Int J Qual Methods. 2017;16(1):1–13. doi: 10.1177/1609406917733847

[27] Alhojailan MI, Ibrahim M. Thematic analysis: a critical review of its process and evaluation. In: WEI International European Academic Conference Proceedings, 2012. 8–21.

[28] Johanna GEB. “ At least three Vacuum Extractions are possible every day!” A questionnaire study in two hospitals in. 2018.

[29] Crossland N, Kingdon C, Balaam M-C, Betrán AP, Downe S. Women’s, partners’ and healthcare providers’ views and experiences of assisted vaginal birth: a systematic mixed methods review. Reprod Health. 2020;17(1):83. doi: 10.1186/s12978-020-00915-w 32487226 PMC7268509

[30] Svelato A, Carabaneanu A, Sergiampietri C, Mannella P, D’Avino S, De Luca C, et al. “To get the baby out off the hook”: a prospective, longitudinal, multicenter, observational study about decision making in vacuum-assisted operative vaginal delivery. BMC Pregnancy Childbirth. 2022;22(1):128. doi: 10.1186/s12884-022-04440-5 35172781 PMC8848824

