# Supplementary material for: Understanding the underuse: Barriers to vacuum-assisted birth among clinicians in Tanzania: An exploratory qualitative study
Source: PLoS One. 2026 Jul 27;21(7):e0354738. doi: 10.1371/journal.pone.0354738 (PMC13405120; doi:10.1371/journal.pone.0354738)
Supplement: S3 Text — (PDF) [file pone.0354738.s003.pdf]

EXCEPTS FROM TRANSCRIPTS

A. Organizational Barriers

Malfunctioning and inaccessible medical equipment

> "*Here in the labor ward, we don't have many tools. However, I believe that we would benefit from having much of this equipment available to us if we approach these situations positively. As it currently stands, we only have one tool." (Resident, 6 years of experience IDI 2*)

> *"The availability of equipment has been a persistent challenge in [hospital], especially in the labor ward. The vacuum cups often fail to grasp the baby's head properly, and the pumps are damaged. However, the process for locating and replacing this equipment is ineffective. Sometimes, the items are simply unavailable in the ward, and replacement depends on the hospital's procurement system, which can be slow and unreliable." (Specialist, 18 years of experience, IDI 8)*

> *"…Additionally, because of the poor condition of our equipment, the cup tends to fall out when we take out the baby." (Specialist, 21 years of experience, IDI 6)*

> *"… For instance, when you are using a vacuum to deliver a baby and need to change the cup, you discover that it is too small for the baby's head and that it may take a while to find a larger one and might never find the proper size"* (*Resident, 9 years of experience* IDI 3)

> *"I can say that these vacuum devices vary quite a bit, so sometimes you may come across a device that you're not used to using for assisting with delivery. At times, you find that the cup is too small. Therefore, I would suggest that they provide a wider range of sizes to enable us to perform the procedure more effectively."*(*Resident, 12 years of experience* IDI 10)

Team Dynamics in deciding when to use VAB

> "*There are conflicting ideas among ourselves; for example, you may decide to help the client by using the vacuum, but another person may say, "No, let's not help her." Fearing*

that the child's outcome will be poor and that you will be held responsible, you may be asked why you did not send her for a caesarian section sooner…" (Specialist, 18 years of experience, IDI 8)

"*There may be a few differences between the doctor and the nurse while exchanging ideas and their views because each has unique experiences and knowledge, but the doctor makes the final decision" (Resident, 9 years of experience, IDI 11)*

"Several SOPs have been posted, such as one for eclampsia. If we post for vacuum delivery, we will all be aware very well that when I see this mother needs this service one hundred percent, and our decision becomes uniform, we know what to do better than waiting for another person to come and make decisions, which in turn delays the provision of services" (Resident, 6 years of experience, IDI 2)

"*…There may be a vacuum case, but they tend to call in special persons so and so to attend to the client; as a result, you realize that the same individuals are managing the issue over and over, leaving others to be observers rather than take action." (Resident, 6 years of experience, IDI 2)*

Limited accessibility and utilization of VAB guidelines and SOPs

"We can say that the guidelines are available, but it depends. Within the clinical team, there are different cadres—doctors, nurses, and nurse attendants. So, you may find that some aspects are more accessible or understandable to doctors because of their level of experience, making it easier for them to comprehend the guidelines. Nurses may also understand them, but for others, such as attendants, they might not be as exposed to certain information—like how to access or implement the guidelines—making it more difficult for them." (Resident, 9 years of experience, IDI 11)

"…as far as I can tell, there are no posted instructions or standard operating procedures (SOPs) in the ward on when and how to perform a vacuum delivery." (Specialist, 15 years of experience, IDI 5)

"The existing SOPs should be posted; they are not too many. Right now, they're just on paper, but if we display them, even someone who has forgotten can refer to them—just like we display guidelines for eclampsia and other conditions. So, in cases where someone needs assistance during delivery, it should show where to place the cup, how many times to pull, and so on. These should also be displayed in the maternity ward." (Specialist, 16 years of experience, IDI 9)

B. Individual barriers

Apprehension regarding adverse outcomes following VAB

"…[Clinicians] are hesitant to use VAB because, if used incorrectly, it could tear the woman's cervix, causing severe bleeding (PPH) and other serious medical conditions..." (Specialist, 15 years of experience, IDI 5)

"…Even when it's necessary, our team rarely utilizes the vacuum too frequently, and I believe this is because we're scared of the potential consequences for the mother and the child…" (Resident, 8 years of experience, IDI 4)

"…When using a vacuum, there's a risk of being held accountable. This blame culture has made doctors reluctant to utilize vacuums or engage in any related practices since they are unaware of them, which makes them dislike performing VABs as time passes." (Resident, 10 years in experience, IDI 12)

"To be honest doctors, feel that this method will not work and hence prefer not to use a vacuum in assisting the mother since their experience in frequently fail, as a result, rather than performing the procedure, you will find that they immediately prepare the patient for surgery" (Resident, 12 years in experience, IDI 10)

"Any adverse outcome must be documented in a report explaining why the mother died, why the baby died, or why the baby had a low Apgar score. Consequently, clinicians fear being questioned or blamed later for why the baby did not thrive or why the delivery had a poor outcome. This fear of blame is particularly strong among private patients, who

constitute the majority of cases here. This culture of blame has led to widespread reluctance to perform vacuum-assisted births." (Residents, 8 years' experience, IDI 1)

Insufficient skills and experience in performing VAB

"I feel the primary issue might be decision-making—whether to use vacuum or not. Beyond the decision itself, it also involves the clinician's knowledge and confidence in performing the procedure. You find that someone knows they should perform it, but they are not comfortable doing so, possibly because they are not fully familiar with the technique or they fear the potential outcomes" (Specialist, 16 years of experience, IDI 9)

"There are a few experienced staff members in this ward, most of them are nurses, midwives, and specialists who have worked in the labor ward for a long time; nevertheless, there are very few other experienced staff members, such as these young residents" (Specialist, 15 years' experience, IDI 5)

"I can say that there are two main reasons why a clinician might not be able to use a vacuum, either they have never used it before or they lack the confidence to use it, which could result in them being unable to use it even if all the indicators are present" (Specialist, 21 years' experience, IDI 7)

"…although there aren't many cases, I believe that we should provide time for practice by utilizing manikins, we might schedule time, perhaps once a month, to gather clinicians, go to simulation rooms, and demonstrate with Mankins, and by doing this periodically, we can develop our skills and improve as a team" (Resident, 8 years of experience, IDI 4).

"What is required is a personal effort from the clinicians themselves and must be able to set aside extra time to read through the guidelines and find time to come into labor ward to learn what is going on and acquire more experience" (Resident, 12 years' experience, IDI 10)

"… as doctors, we have many duties in the ward and elsewhere, and we have very little time in labor. As a result, you don't have a lot of opportunity to practice in real life because there isn't much equipment available." (Specialist, 21 years of experience, IDI 6)
