# Supplementary material for: Understanding the underuse: Barriers to vacuum-assisted birth among clinicians in Tanzania: An exploratory qualitative study
Source: PLoS One. 2026 Jul 27;21(7):e0354738. doi: 10.1371/journal.pone.0354738 (PMC13405120; doi:10.1371/journal.pone.0354738)
Supplement: Attachment — Submitted filename: PONE-D-25-41107 Vacuum deliveries-Reviewer report-20260110.docx [file pone.0354738.s005.docx]

**PLOS One-D-25-41107 Understanding the underuse: Barriers to Vacuum-assisted birth among clinicians in Tanzania: An exploratory qualitative study**

Review report: Dr Sheila E Clow

Thank you for the opportunity to review this study.

Using the criteria for publication my responses are as follows

1. [The study presents the results of original research.](https://journals.plos.org/plosone/s/criteria-for-publication#loc-1)

The study presents results of original research local to Tanzania, with relevance to low resource settings

2. [Results reported have not been published elsewhere](https://journals.plos.org/plosone/s/criteria-for-publication#loc-2).

To my knowledge these results have not been published elsewhere

3. [Experiments, statistics, and other analyses are performed to a high technical standard and are described in sufficient detail.](https://journals.plos.org/plosone/s/criteria-for-publication#loc-3)

The study employed a descriptive qualitative approach so the quantitative research requirements are not relevant. The article describes the methods used. There are some areas that can be strengthened which are outlines in the comments to the author and to the editor.

4. [Conclusions are presented in an appropriate fashion and are supported by the data](https://journals.plos.org/plosone/s/criteria-for-publication#loc-4).

Yes

5. [The article is presented in an intelligible fashion and is written in standard English](https://journals.plos.org/plosone/s/criteria-for-publication#loc-5).

Yes. There are some grammatical / typos that need to be corrected. These do not detract from the presentation and understanding of the article. These are highlighted in the comments to the editor

6. [The research meets all applicable standards for the ethics of experimentation and research integrity](https://journals.plos.org/plosone/s/criteria-for-publication#loc-6).

Experimentation is not relevant in this study. Ethical requirements are presented. There are further ethical considerations that are not explicitly described which are included in the comments to the author, and which should be addressed.

7. [The article adheres to appropriate reporting guidelines and community standards for data availability](https://journals.plos.org/plosone/s/criteria-for-publication#loc-7).

Yes
