# Supplementary material for: Understanding the underuse: Barriers to vacuum-assisted birth among clinicians in Tanzania: An exploratory qualitative study
Source: PLoS One. 2026 Jul 27;21(7):e0354738. doi: 10.1371/journal.pone.0354738 (PMC13405120; doi:10.1371/journal.pone.0354738)
Supplement: Attachment — Submitted filename: PONE-D-25-41107_SB.docx [file pone.0354738.s004.docx]

**Review: PONE-D-25-41107**
**Title:** Understanding the Underuse: Barriers to Vacuum-Assisted Birth Among Clinicians in Tanzania: An Exploratory Qualitative Study

**Overall Assessment**

This is a clinically relevant qualitative study with relatively good methodology that addresses an important gap in maternal healthcare in low-resource settings. The findings might have practical implications in Tanzania and similar contexts. However, some areas might require revisions to meet the publication standards of *PLOS ONE*.

There are few comments/suggestions and few queries raised for clarification.

**Introduction**

Inclusion of recent Tanzanian or East African data on CS and VAB rates to contextualize the problem to make the rationale stronger.

**Methods**

**Data collection tools and procedure**

Are the experiences of residents and experts considered from the time participants had undertaken their medical profession or since they started their medical study? It would be fair to make it clear for the readers.

Page no 5 line 96, ‘existing signatures (include references)’ meaning not clear.

If the review of the past study ensures relevant themes, then it was deductive approach. You did not use data driven theme then. Is this what you mean? Please mention what approach was used.

Validation of language translation not clearly mentioned. When the tool was translated into local language how was it ensured that the meaning did not change after translation.

- Mention how were the cultural relevance ensured? What changes were made in the English version to adapt the local culture?
- Provide more detail on how the guide was developed, piloted, and refined. Include sample questions in supplementary materials.

**Results**

The author should made clear who is the person directly involved in VAB in this study. If it is residents or obs/gynecologists, it’s ok. It if is done by trained nursing staff, then this study tells only half part of the story. Author reports nursing attendant should also be exposed to VAB guidelines. If they are not directly involved in the procedure, in what way they can influence the use of VAB should be clearly mentioned.

**Theme ‘Limited accessibility’ Page no 240**

It is known that guidelines for VAB were not available in the ward which made the decision making difficult for the residents. Are not they oriented to all birthing procedure during their medical study or at least before working in the gyne/obs department? Has this been explored? In this study whether if in any form they get orientation regarding the procedures.

Page no 251-254, attendants might not be exposed to information such as how to access or implement the guidelines. How does patient attendant help in VAB even if the attendant have access to guidelines. Does this attendant mean someone from the birthing crew? Who are these nurse attendants?

Page 349, 350 meaning not clear in the quote.

Some of the subthemes can be merged which has been repeated in multiple sections, such as fear of blame etc. to avoid repetition.

**Discussion**

- Discussion regarding how Tanzanian barriers are similar to or different from those in other settings is limited.
