# Supplementary material for: Understanding the underuse: Barriers to vacuum-assisted birth among clinicians in Tanzania: An exploratory qualitative study
Source: PLoS One. 2026 Jul 27;21(7):e0354738. doi: 10.1371/journal.pone.0354738 (PMC13405120; doi:10.1371/journal.pone.0354738)
Supplement: Attachment — Submitted filename: Response to Reviewewers.docx [file pone.0354738.s007.docx]

**Research Title:** **Understanding the Underuse: Barriers to Vacuum-Assisted Birth among Clinicians in Tanzania: An exploratory qualitative study**

| **SN** | **Reviewer’s comments** | **Authors’ Response** |
| --- | --- | --- |
|  | **Comments from Reviewer 1** | |
| **1** | **Introduction**  Inclusion of recent Tanzanian or East African data on CS and VAB rates to contextualize the problem to make the rationale stronger. | Thank you for this valuable suggestion. We have strengthened the Introduction by incorporating recent evidence on caesarean section and vacuum-assisted birth rates from Tanzania and East Africa. These data highlight the increasing use of caesarean section alongside the persistently low utilization of vacuum-assisted birth, thereby providing a stronger regional context and reinforcing the rationale for investigating the underuse of vacuum-assisted birth among clinicians in Tanzania (Introduction, pages 3–4, lines 52–70). |
| **2** | **Data collection tools and procedure**  Are the experiences of residents and experts considered from the time participants had undertaken their medical profession or since they started their medical study? It would be fair to make it clear to the readers. | Thank you for asking. We have improved the description of participants recruited into the study to include their experience as medical specialists, as well as those who had completed one year of residency and are pursuing specialist training in obstetrics. |
| **3** | Page no 5 line 96, ‘existing signatures (include references)’ meaning not clear. | Thank you for your observation. We have reviewed the statement and included the appropriate words “existing literature.” |
| **4** | If the review of the past study ensures relevant themes, then it was a deductive approach. You did not use data driven theme then. Is this what you mean? Please mention what approach was used. | Thank you for the comment. The review of the past literature was done to identify domains related to VAB use and hence structure interview questions rather than an analysis of data. Themes were developed inductively from data. |
| **5** | Mention how were the cultural relevance ensured? What changes were made in the English version to adapt the local culture? | The tool was developed in the English language; however, researchers and participants are native Swahili speakers. Translation of the tool to the Kiswahili language and adjustment of the terms to the local clinical environment ensured that the tool aligns with the communication culture. This section has been improved by adding more context. |
| **6** | Provide more detail on how the guide was developed, piloted, and refined. Include sample questions in supplementary materials. | Thank you for the comment. More details have been provided to clarify the development of the tool. |
|  | **Results** |  |
| **7** | The author should make clear who is the person directly involved in VAB in this study. If it is residents or obs/gynecologists, it’s ok. If it is done by trained nursing staff, then this study tells only half part of the story. Author reports nursing attendant should also be exposed to VAB guidelines. If they are not directly involved in the procedure, in what way they can influence the use of VAB should be clearly mentioned. | We agree that attendants are not directly involved in performing vacuum-assisted birth (VAB). They were mentioned alongside nurses by a resident during the interview in the context of comparing their understanding of the guideline. As attendants and nurses were not part of the study population, we have revised this section by replacing the quotation with more appropriate excerpts that pertain exclusively to clinicians. |
| **8** | Theme ‘Limited accessibility’ Page no 240  It is known that guidelines for VAB were not available in the ward which made the decision making difficult for the residents. Are not they oriented to all birthing procedure during their medical study or at least before working in the gyne/obs department? Has this been explored? In this study whether if in any form they get orientation regarding the procedures. | Thank you for your comment. Clinicians are typically introduced to all major birthing procedures during their formal medical training. However, within the context of this study, participants, particularly those with limited clinical experience, expressed that the availability of clinical guidelines in the labour ward would have supported decision-making and enhanced their confidence in performing vacuum-assisted birth (VAB) successfully. |
| **9** | Page no 251-254, attendants might not be exposed to information such as how to access or implement the guidelines. How does patient attendant help in VAB even if the attendant have access to guidelines. Does this attendant mean someone from the birthing crew? Who are these nurse attendants? | Thank you for your comment. Attendants are not directly involved in performing vacuum-assisted birth (VAB) and therefore do not influence guideline implementation. Their role is limited to supportive tasks, such as transporting and cleaning equipment. As they were not part of the study population, references to them have been omitted from the manuscript. |
| **10** | Page 349, 350, meaning not clear in the quote. | The quotation has been replaced with a clearer excerpt, and additional explanation has been incorporated to enhance clarity and contextual understanding. |
| **11** | Some of the subthemes can be merged, which have been repeated in multiple sections, such as fear of blame, etc. to avoid repetition. | Thank you for your comment. We have checked and rectified |
|  | **Discussion** |  |
| **12** | Discussion regarding how Tanzanian barriers are similar to or different from those in other settings is limited. | Thank you for your comment. We note that our findings have been compared with those from other studies (page 21, lines 425–427; page 22, lines 442-445; and page 23, lines 465-468). |
|  |  |  |
|  | **Comments from Reviewer 2** | |
| **1** | Line 52: (Despite its known benefits, VAB the utilization of VAB remains…) This sentence will have to be checked to avoid repetition of terms. | This sentence has been revised to remove the repeating word ‘VAB’ and improve clarity. |
| **2** | Line 64: (This utilization may be contributing) This could be changed to “This underutilization may…” | We appreciate your suggestion. The statement has been changed to “This underutilization may be contributing to the increasing rates of CS deliveries in the country.” |
| **3** | Lines 397-401: (In some instances, the use of inappropriate sizes of vacuum extracted cups…) and (Inappropriate sizes of vacuum extractor cups have resulted in slippage during procedures…). These two sentences appear to be communicating the same point. It will benefit the manuscript if the authors can make a single statement from both or keep one | Thank you for the comment. We agree with you and have therefore retained only one statement to avoid redundancy and ensure clarity. |
| **4** | Lines 408-409: (These results are consistent with studies conducted in other LMICs…). It would be more appropriate to be more specific with the names of these LMICs. | Thank you for the comment. The LMICs referred to in this section are those represented in the secondary data, which were analyzed and reported in aggregate (Latin America, sub-Saharan Africa, and Asia). These regions have now been explicitly specified in the manuscript for clarity. |
| **5** | Lines 433-435: (Similar concerns have been reported in other African countries…). It would be more appropriate to be more specific with the names of these African countries. | The statement has been revised, and specific countries have been highlighted. |
| **6** | Additionally, including the data collection tool as a supplementary file would benefit both the readers of the manuscript and improve its perspective. | Thank you for the comment. The in-depth interview guide has been added as a supplementary file. |
| **7** | The study makes a good contribution to the field and would benefit from proofreading to correct some grammatical errors. | Proofreading has been done to correct grammatical errors. |
|  |  |  |
|  | **Comments from Reviewer 3** | |
| **1** | In understanding the context of the research (which relates to transferability), it would be helpful to describe for the international readership the type (e.g. Kiwi, Silc-cup) and composition (e.g. metal, silicone) of the vacuum systems (e.g. piped or hand-pump operated compressed air) available. | Thank you for this helpful suggestion. The Methods section has been amended to include details regarding the vacuum extraction apparatus utilized at the study site. We have detailed the type and substance of the vacuum cup and the vacuum generation mechanism utilized in the hospital. This supplementary information offers crucial clinical context for international readers who may lack familiarity with the instruments utilized for vacuum-assisted delivery in this setting. |
| **2** | **Clarifications** Please clarify and define early in the article the clinical specialists included in this study. There is frequent generic reference made to skilled health care providers, and later on (line 248-54) mention is made of nurses, midwives, and nurse attendants. It is unclear why these categories of health care providers are mentioned when the focus appears to be on the barriers experienced by medical clinicians. Are midwives or nurses taught and licensed to undertake vacuum deliveries? | Thank you for your comment. We have added explicit descriptions about the participants of this study. Nurses and midwives are not part of this study, regardless of being trained to perform VAB. Therefore, any information in reference to health care providers has been reviewed and clarified. |
| **3** | Please clarify what is meant by clinical experience. Does this include clinical experience as an undergraduate medical student or only after qualification. Alternatively, does this relate specifically to obstetric clinical experience? | Clinical experience in this study refers to working experience after qualification. Undergraduate medical training was not explored in this study. We have clarified this in the participant and recruitment section. |
| **4** | As this is a qualitative study it would be prudent to disclose the authors’ experience / perspective of this clinical situation as this is germane to their research conduct and interpretation. • The article would be strengthened by including information about the development of the semi-structured interview guide. It is unclear what informed the questions, i.e., what was the theoretical or conceptual base? This level of detail is relevant for the credibility aspect of trustworthiness. What is mentioned in lines 95/6 is “The tool was informed by a review of existing signatures (include references) to ensure relevant themes on barriers to VAB use”. It is not clear what is meant by a review of existing signatures (include references)”. | Thank you very much for your comment. The development of the interview guide has been further explained. The guide was designed through a structured literature review, and the socioecological model guided the structure and organization of questions. Moreover, line 95/6 has been reviewed and the appropriate terms incorporated. |
| **5** | Research rigour / Trustworthiness. It would strengthen the article to explicitly include “transferability” and to mention if member checking was done to enhance confirmability. | Thank you for your comment. To improve transferability, we have elucidated the contextual attributes of the study environment, encompassing facility level, delivery volume, VAB rates, and organizational context. We also supplied comprehensive participant characteristics, including professional cadre (expert obstetricians versus residents), job experience, and additional recruitment criteria. These descriptions enable readers to evaluate the relevance of findings to analogous clinical settings.  Formal member checking was not conducted due to logistical limitations and the restricted availability of clinicians. Confirmability was improved through methodological triangulation within the study team, and independent coding by various researchers, followed by consensus discussions. |
| **6** | **Ethics**  While confidentiality is clearly described there are other ethical concerns • Participants were identified by the ward “incharge”, after which the researcher approached them in person. Did the “incharge” ask the potential participants if the researchers could approach them? This distinction is important in terms of the autonomy of the individuals concerned who were in the employ of the hospital. | Thank you for your observation. In this study, the role of the in-charge was to identify potential participants and produce a list, which was given to the researchers. Then the researcher was the one who obtained consent from participants, and all participants were free to choose whether to participate or not. The in-charge did not ask participants anything about being approached by the researcher. |
| **7** | What was the employment relationship between the participants and the researchers? Given that the participants were employees in the hospital where the study was being conducted, was there a sense of obligation to participate (undue coercion?)? What was the power dynamic between the participants and the employer and/or the researcher? Was there a possibility that they might feel that their participation was necessary to ensure that their employment status was not jeopardised? | The relationship between the researchers and participants was limited strictly to the conduct of the study. All researchers were affiliated with Muhimbili University of Health and Allied Sciences, while participants were employees of Muhimbili National Hospital. Although both institutions share the name “Muhimbili,” they operate independently under distinct management structures, despite being located adjacent to one another. |
| **8** | Author contributions. Please clarify which of the authors conducted the interviews. The article indicates that a midwife was a co-facilitator, but it is not clear who was the lead interviewer. It is not clear if the midwife co-facilitator (information gatherer/data collector) is included in the authorship or acknowledgements. Please clarify this contribution. | Thank you for the comment. We have updated the author's contribution. The RA has also been included as a Co-author because he participated in curating data and creating the initial draft of the manuscript, which warrants authorship. |
|  |  |  |
