# Supplementary material for: Understanding the underuse: Barriers to vacuum-assisted birth among clinicians in Tanzania: An exploratory qualitative study
Source: PLoS One. 2026 Jul 27;21(7):e0354738. doi: 10.1371/journal.pone.0354738 (PMC13405120; doi:10.1371/journal.pone.0354738)
Supplement: Peer review history

# Author comment: Author response to Decision Letter 0

**DOI:** 10.1371/journal.pone.0354738.r001

**Transfer Alert**

This paper was transferred from another journal. As a result, its full editorial history (including decision letters, peer reviews and author responses) may not be present.

16 Aug 2025

# Peer review: Decision Letter 0

**DOI:** 10.1371/journal.pone.0354738.r002

**Authors:** Hannah Tappis

18 Jan 2026

PONE-D-25-41107

Understanding the Underuse: Barriers to Vacuum-Assisted Birth Among Clinicians in Tanzania: An Exploratory Qualitative Study

PLOS One

Dear Dr. Chikwala,

Thank you for submitting your manuscript to PLOS ONE. After careful consideration, we feel that it has merit but does not fully meet PLOS ONE’s publication criteria as it currently stands. Therefore, we invite you to submit a revised version of the manuscript that addresses the points raised during the review process.

Please submit your revised manuscript by Mar 04 2026 11:59PM. If you will need more time than this to complete your revisions, please reply to this message or contact the journal office at plosone@plos.org. When you're ready to submit your revision, log on to https://www.editorialmanager.com/pone/ and select the 'Submissions Needing Revision' folder to locate your manuscript file.

Please include the following items when submitting your revised manuscript:

- A letter that responds to each point raised by the academic editor and reviewer(s). You should upload this letter as a separate file labeled 'Response to Reviewers'.
- A marked-up copy of your manuscript that highlights changes made to the original version. You should upload this as a separate file labeled 'Revised Manuscript with Track Changes'.
- An unmarked version of your revised paper without tracked changes. You should upload this as a separate file labeled 'Manuscript'.

If you would like to make changes to your financial disclosure, please include your updated statement in your cover letter. Guidelines for resubmitting your figure files are available below the reviewer comments at the end of this letter.

If applicable, we recommend that you deposit your laboratory protocols in protocols.io to enhance the reproducibility of your results. Protocols.io assigns your protocol its own identifier (DOI) so that it can be cited independently in the future. For instructions see: https://journals.plos.org/plosone/s/submission-guidelines#loc-laboratory-protocols. Additionally, PLOS ONE offers an option for publishing peer-reviewed Lab Protocol articles, which describe protocols hosted on protocols.io. Read more information on sharing protocols at https://plos.org/protocols?utm_medium=editorial-email&utm_source=authorletters&utm_campaign=protocols.

We look forward to receiving your revised manuscript.

Kind regards,

Hannah Tappis, DrPH, MPH

Academic Editor

PLOS One

Journal Requirements:

When submitting your revision, we need you to address these additional requirements.

1.Please ensure that your manuscript meets PLOS ONE's style requirements, including those for file naming. The PLOS ONE style templates can be found at

https://journals.plos.org/plosone/s/file?id=wjVg/PLOSOne_formatting_sample_main_body.pdf and

https://journals.plos.org/plosone/s/file?id=ba62/PLOSOne_formatting_sample_title_authors_affiliations.pdf

2. Your ethics statement should only appear in the Methods section of your manuscript. If your ethics statement is written in any section besides the Methods, please move it to the Methods section and delete it from any other section. Please ensure that your ethics statement is included in your manuscript, as the ethics statement entered into the online submission form will not be published alongside your manuscript.

3. Please include captions for your Supporting Information files at the end of your manuscript, and update any in-text citations to match accordingly. Please see our Supporting Information guidelines for more information: http://journals.plos.org/plosone/s/supporting-information.

4. If the reviewer comments include a recommendation to cite specific previously published works, please review and evaluate these publications to determine whether they are relevant and should be cited. There is no requirement to cite these works unless the editor has indicated otherwise.

[Note: HTML markup is below. Please do not edit.]

Reviewer's Responses to Questions

**Comments to the Author**

1. Is the manuscript technically sound, and do the data support the conclusions?

The manuscript must describe a technically sound piece of scientific research with data that supports the conclusions. Experiments must have been conducted rigorously, with appropriate controls, replication, and sample sizes. The conclusions must be drawn appropriately based on the data presented.

Reviewer #1: Partly

Reviewer #2: Yes

Reviewer #3: Yes

**********

2. Has the statistical analysis been performed appropriately and rigorously?

Reviewer #1: N/A

Reviewer #2: N/A

Reviewer #3: N/A

**********

3. Have the authors made all data underlying the findings in their manuscript fully available?

The PLOS Data policy requires authors to make all data underlying the findings described in their manuscript fully available without restriction, with rare exception (please refer to the Data Availability Statement in the manuscript PDF file). The data should be provided as part of the manuscript or its supporting information, or deposited to a public repository. For example, in addition to summary statistics, the data points behind means, medians and variance measures should be available. If there are restrictions on publicly sharing data—e.g. participant privacy or use of data from a third party—those must be specified.

Reviewer #1: No

Reviewer #2: Yes

Reviewer #3: Yes

**********

4. Is the manuscript presented in an intelligible fashion and written in standard English?

PLOS ONE does not copyedit accepted manuscripts, so the language in submitted articles must be clear, correct, and unambiguous. Any typographical or grammatical errors should be corrected at revision, so please note any specific errors here.

Reviewer #1: Yes

Reviewer #2: Yes

Reviewer #3: Yes

**********

5. Review Comments to the Author

Please use the space provided to explain your answers to the questions above. You may also include additional comments for the author, including concerns about dual publication, research ethics, or publication ethics. (Please upload your review as an attachment if it exceeds 20,000 characters)

Reviewer #1: Dear Author, This is a relevant and fairly good methodological study to explore the maternal health in-depth regarding VAB in LIMC. Here are few suggestions and queries that I think needs to be cleared before the publication.

Strengthen the introduction and discussion.

Clarify and deepen the thematic analysis.

The details of comments and queries are attached as a separate file. Thank you

Reviewer #2: Thank you for the opportunity to review this manuscript. This manuscript addresses an important and underexplored issue in maternal health by examining the barriers to the use of vacuum-assisted birth among clinicians in Tanzania. Given the continued burden of prolonged labor and preventable maternal and neonatal complications in low-resource settings, understanding factors influencing the underuse of evidence-based obstetric interventions is highly relevant. The study’s focus on clinicians’ perspectives and its use of an exploratory qualitative design are appropriate for capturing contextual, system-level, and experiential barriers. The topic aligns well with the scope of PLOS ONE and has the potential to contribute valuable insights to obstetric practice, health systems strengthening, and policy discussions in similar settings.

Below are some comments that, when addressed, could improve the quality of the manuscript:

1. Line 52: (Despite its known benefits, VAB the utilization of VAB remains…) This sentence will have to be checked to avoid repetition of terms.

2. Line 64: (This utilization may be contributing) This could be changed to “This underutilization may…”

3. Lines 397-401: (In some instances, the use of inappropriate sizes of vacuum extracted cups…) and (Inappropriate sizes of vacuum extractor cups have resulted in slippage during procedures…). These two sentences appear to be communicating the same point. It will benefit the manuscript if the authors can make a single statement from both or keep one.

4. Lines 408-409: (These results are consistent with studies conducted in other LMICs…). It would be more appropriate to be more specific with the names of these LMICs.

5. Lines 433-435: (Similar concerns have been reported in other African countries…). It would be more appropriate to be more specific with the names of these African countries.

6. Additionally, including the data collection tool as a supplementary file would benefit both the readers of the manuscript and improve its perspective.

7. The study makes a good contribution to the field and would benefit from proofreading to correct some grammatical errors.

Reviewer #3: Comments to author

This is a worthwhile study, particularly in a low resource setting to determine what barriers exist to using a safe alternative to avoid C/S which carry significant risks (especially in a setting with a 75% C/S rate). In understanding the context of the research (which relates to transferability), it would be helpful to describe for the international readership the type (e.g. Kiwi, Silc-cup) and composition (e.g. metal, silicone) of the vacuum systems (e.g. piped or hand-pump operated compressed air) available.

Clarifications

• Please clarify and define early in the article the clinical specialists included in this study. There is frequent generic reference made to skilled health care providers, and later on (line 248-54) mention is made of nurses, midwives, and nurse attendants. It is unclear why these categories of health care providers are mentioned when the focus appears to be on the barriers experienced by medical clinicians. Are midwives or nurses taught and licensed to undertake vacuum deliveries?

• Please clarify what is meant by clinical experience. Does this include clinical experience as an undergraduate medical student or only after qualification. Alternatively does this relate specifically to obstetric clinical experience?

As this is a qualitative study it would be prudent to disclose the authors’ experience / perspective of this clinical situation as this is germane to their research conduct and interpretation.

• The article would be strengthened by including information about the development of the semi-structured interview guide. It is unclear what informed the questions, i.e. what was the theoretical or conceptual base? This level of detail is relevant for the credibility aspect of trustworthiness. What is mentioned in lines 95/6 is “The tool was informed by a review of existing signatures (include references) to ensure relevant themes on barriers to VAB use”. It is not clear what is meant by a review of existing signatures (include references)”.

• Research rigour / Trustworthiness. It would strengthen the article to explicitly include “transferability” and to mention if member checking was done to enhance confirmability.

Discussion. This is well founded on the information gathered (data). However,

• There is no mention of the knowledge foundation of the participants (to address lack of confidence in decision-making).

• While it is understood that the context (equipment availability and functionality, and clinical skills) is not conducive to successful VAB, it appears that some of the participants believed that VAB was harmful per se, despite long standing evidence to the contrary.

• While mention is made of lack of agreed clinical guidelines and SOPs, and challenging interpersonal relationships among the labour ward staff, the article is silent on the apparent lack of clinical leadership which is required to manage such dynamics and processes.

Ethics. While confidentiality is clearly described there are other ethical concerns

• Participants were identified by the ward “incharge”, after which the researcher approached them in person. Did the “incharge” ask the potential participants if the researchers could approach them? This distinction is important in terms of the autonomy of the individuals concerned who were in the employ of the hospital.

• What was the employment relationship between the participants and the researchers? Given that the participants were employees in the hospital where the study was being conducted, was there a sense of obligation to participate (undue coercion?), what was the power dynamic between the participants and the employer and/or the researcher? Was there a possibility that they might feel that their participation was necessary to ensure that their employment status was not jeopardised?

References. A thorough check of the reference list required to ensure that references are complete and correct, specifically 1, 4, 5, 7, 12, 18, 24, 26

Author contributions. Please clarify which of the authors conducted the interviews. The article indicates that a midwife was a co-facilitator, but it is not clear who was the lead interviewer. It is not clear if the midwife co-facilitator (information gatherer / data collector) is included in the authorship or acknowledgements. Please clarify this contribution.

I wish you well in any follow-up quality improvement initiative to strengthen this aspect of clinical practice for the benefit of mothers and babies in your setting.

**********

6. PLOS authors have the option to publish the peer review history of their article (what does this mean?). If published, this will include your full peer review and any attached files.

If you choose “no”, your identity will remain anonymous but your review may still be made public.

**Do you want your identity to be public for this peer review?**  For information about this choice, including consent withdrawal, please see our Privacy Policy.

Reviewer #1: **Yes:**  Dr. Sailesh Bhattarai

Reviewer #2: No

Reviewer #3: No

**********

[NOTE: If reviewer comments were submitted as an attachment file, they will be attached to this email and accessible via the submission site. Please log into your account, locate the manuscript record, and check for the action link "View Attachments". If this link does not appear, there are no attachment files.]

To ensure your figures meet our technical requirements, please review our figure guidelines: https://journals.plos.org/plosone/s/figures

You may also use PLOS’s free figure tool, NAAS, to help you prepare publication quality figures: https://journals.plos.org/plosone/s/figures#loc-tools-for-figure-preparation.

NAAS will assess whether your figures meet our technical requirements by comparing each figure against our figure specifications.

Attachment Submitted filename: PONE-D-25-41107_SB.docx

Attachment Submitted filename: PONE-D-25-41107 Vacuum deliveries-Reviewer report-20260110.docx

# Author comment: Author response to Decision Letter 1

**DOI:** 10.1371/journal.pone.0354738.r003

7 Jul 2026

Comments from Reviewer 1

1 Introduction

Inclusion of recent Tanzanian or East African data on CS and VAB rates to contextualize the problem to make the rationale stronger.

Authors’ Response

Thank you for this valuable suggestion. We have strengthened the Introduction by incorporating recent evidence on caesarean section and vacuum-assisted birth rates from Tanzania and East Africa. These data highlight the increasing use of caesarean section alongside the persistently low utilization of vacuum-assisted birth, thereby providing a stronger regional context and reinforcing the rationale for investigating the underuse of vacuum-assisted birth among clinicians in Tanzania (Introduction, pages 3–4, lines 52–70).

2 Data collection tools and procedure

Are the experiences of residents and experts considered from the time participants had undertaken their medical profession or since they started their medical study? It would be fair to make it clear to the readers.

Authors’ Response

Thank you for asking. We have improved the description of participants recruited into the study to include their experience as medical specialists, as well as those who had completed one year of residency and are pursuing specialist training in obstetrics.

3 Page no 5 line 96, ‘existing signatures (include references)’ meaning not clear. Authors’ Response

Thank you for your observation. We have reviewed the statement and included the appropriate words “existing literature.”

4 If the review of the past study ensures relevant themes, then it was a deductive approach. You did not use data driven theme then. Is this what you mean? Please mention what approach was used.

Authors’ Response

Thank you for the comment. The review of the past literature was done to identify domains related to VAB use and hence structure interview questions rather than an analysis of data. Themes were developed inductively from data.

5 Mention how were the cultural relevance ensured? What changes were made in the English version to adapt the local culture?

Authors’ Response

The tool was developed in the English language; however, researchers and participants are native Swahili speakers. Translation of the tool to the Kiswahili language and adjustment of the terms to the local clinical environment ensured that the tool aligns with the communication culture. This section has been improved by adding more context.

6 Provide more detail on how the guide was developed, piloted, and refined. Include sample questions in supplementary materials.

Authors’ Response

Thank you for the comment. More details have been provided to clarify the development of the tool.

Results

7 The author should make clear who is the person directly involved in VAB in this study. If it is residents or obs/gynecologists, it’s ok. If it is done by trained nursing staff, then this study tells only half part of the story. Author reports nursing attendant should also be exposed to VAB guidelines. If they are not directly involved in the procedure, in what way they can influence the use of VAB should be clearly mentioned.

Authors’ Response

We agree that attendants are not directly involved in performing vacuum-assisted birth (VAB). They were mentioned alongside nurses by a resident during the interview in the context of comparing their understanding of the guideline. As attendants and nurses were not part of the study population, we have revised this section by replacing the quotation with more appropriate excerpts that pertain exclusively to clinicians.

8 Theme ‘Limited accessibility’ Page no 240

It is known that guidelines for VAB were not available in the ward which made the decision making difficult for the residents. Are not they oriented to all birthing procedure during their medical study or at least before working in the gyne/obs department? Has this been explored? In this study whether if in any form they get orientation regarding the procedures.

Authors’ Response

Thank you for your comment. Clinicians are typically introduced to all major birthing procedures during their formal medical training. However, within the context of this study, participants, particularly those with limited clinical experience, expressed that the availability of clinical guidelines in the labour ward would have supported decision-making and enhanced their confidence in performing vacuum-assisted birth (VAB) successfully.

9 Page no 251-254, attendants might not be exposed to information such as how to access or implement the guidelines. How does patient attendant help in VAB even if the attendant have access to guidelines. Does this attendant mean someone from the birthing crew? Who are these nurse attendants?

Authors’ Response

Thank you for your comment. Attendants are not directly involved in performing vacuum-assisted birth (VAB) and therefore do not influence guideline implementation. Their role is limited to supportive tasks, such as transporting and cleaning equipment. As they were not part of the study population, references to them have been omitted from the manuscript.

10 Page 349, 350, meaning not clear in the quote.

Authors’ Response

The quotation has been replaced with a clearer excerpt, and additional explanation has been incorporated to enhance clarity and contextual understanding.

11 Some of the subthemes can be merged, which have been repeated in multiple sections, such as fear of blame, etc. to avoid repetition.

Authors’ Response

Thank you for your comment. We have checked and rectified

Discussion

12 Discussion regarding how Tanzanian barriers are similar to or different from those in other settings is limited.

Authors’ Response

Thank you for your comment. We note that our findings have been compared with those from other studies (page 21, lines 425–427; page 22, lines 442-445; and page 23, lines 465-468).

Comments from Reviewer 2

1 Line 52: (Despite its known benefits, VAB the utilization of VAB remains…) This sentence will have to be checked to avoid repetition of terms.

Authors’ Response

This sentence has been revised to remove the repeating word ‘VAB’ and improve clarity.

2 Line 64: (This utilization may be contributing) This could be changed to “This underutilization may…”

Authors’ Response

We appreciate your suggestion. The statement has been changed to “This underutilization may be contributing to the increasing rates of CS deliveries in the country.”

3 Lines 397-401: (In some instances, the use of inappropriate sizes of vacuum extracted cups…) and (Inappropriate sizes of vacuum extractor cups have resulted in slippage during procedures…). These two sentences appear to be communicating the same point. It will benefit the manuscript if the authors can make a single statement from both or keep one

Authors’ Response

Thank you for the comment. We agree with you and have therefore retained only one statement to avoid redundancy and ensure clarity.

4 Lines 408-409: (These results are consistent with studies conducted in other LMICs…). It would be more appropriate to be more specific with the names of these LMICs.

Authors’ Response

Thank you for the comment. The LMICs referred to in this section are those represented in the secondary data, which were analyzed and reported in aggregate (Latin America, sub-Saharan Africa, and Asia). These regions have now been explicitly specified in the manuscript for clarity.

5 Lines 433-435: (Similar concerns have been reported in other African countries…). It would be more appropriate to be more specific with the names of these African countries.

Authors’ Response

The statement has been revised, and specific countries have been highlighted.

6 Additionally, including the data collection tool as a supplementary file would benefit both the readers of the manuscript and improve its perspective.

Authors’ Response

Thank you for the comment. The in-depth interview guide has been added as a supplementary file.

7 The study makes a good contribution to the field and would benefit from proofreading to correct some grammatical errors.

8 Authors’ Response

Proofreading has been done to correct grammatical errors.

Comments from Reviewer 3

1 In understanding the context of the research (which relates to transferability), it would be helpful to describe for the international readership the type (e.g. Kiwi, Silc-cup) and composition (e.g. metal, silicone) of the vacuum systems (e.g. piped or hand-pump operated compressed air) available.

Authors’ Response

Thank you for this helpful suggestion. The Methods section has been amended to include details regarding the vacuum extraction apparatus utilized at the study site. We have detailed the type and substance of the vacuum cup and the vacuum generation mechanism utilized in the hospital. This supplementary information offers crucial clinical context for international readers who may lack familiarity with the instruments utilized for vacuum-assisted delivery in this setting.

2 Clarifications

Please clarify and define early in the article the clinical specialists included in this study. There is frequent generic reference made to skilled health care providers, and later on (line 248-54) mention is made of nurses, midwives, and nurse attendants. It is unclear why these categories of health care providers are mentioned when the focus appears to be on the barriers experienced by medical clinicians. Are midwives or nurses taught and licensed to undertake vacuum deliveries?

Authors’ Response

Thank you for your comment. We have added explicit descriptions about the participants of this study. Nurses and midwives are not part of this study, regardless of being trained to perform VAB. Therefore, any information in reference to health care providers has been reviewed and clarified.

3 Please clarify what is meant by clinical experience. Does this include clinical experience as an undergraduate medical student or only after qualification. Alternatively, does this relate specifically to obstetric clinical experience?

Authors’ Response

Clinical experience in this study refers to working experience after qualification. Undergraduate medical training was not explored in this study. We have clarified this in the participant and recruitment section.

4 As this is a qualitative study it would be prudent to disclose the authors’ experience / perspective of this clinical situation as this is germane to their research conduct and interpretation.

• The article would be strengthened by including information about the development of the semi-structured interview guide. It is unclear what informed the questions, i.e., what was the theoretical or conceptual base? This level of detail is relevant for the credibility aspect of trustworthiness. What is mentioned in lines 95/6 is “The tool was informed by a review of existing signatures (include references) to ensure relevant themes on barriers to VAB use”. It is not clear what is meant by a review of existing signatures (include references)”.

Authors’ Response

Thank you very much for your comment. The development of the interview guide has been further explained. The guide was designed through a structured literature review, and the socioecological model guided the structure and organization of questions. Moreover, line 95/6 has been reviewed and the appropriate terms incorporated.

5 Research rigour / Trustworthiness. It would strengthen the article to explicitly include “transferability” and to mention if member checking was done to enhance confirmability.

Authors’ Response

Thank you for your comment. To improve transferability, we have elucidated the contextual attributes of the study environment, encompassing facility level, delivery volume, VAB rates, and organizational context. We also supplied comprehensive participant characteristics, including professional cadre (expert obstetricians versus residents), job experience, and additional recruitment criteria. These descriptions enable readers to evaluate the relevance of findings to analogous clinical settings.

Formal member checking was not conducted due to logistical limitations and the restricted availability of clinicians. Confirmability was improved through methodological triangulation within the study team, and independent coding by various researchers, followed by consensus discussions.

6 Ethics

While confidentiality is clearly described there are other ethical concerns

• Participants were identified by the ward “incharge”, after which the researcher approached them in person. Did the “incharge” ask the potential participants if the researchers could approach them? This distinction is important in terms of the autonomy of the individuals concerned who were in the employ of the hospital.

Authors’ Response

Thank you for your observation. In this study, the role of the in-charge was to identify potential participants and produce a list, which was given to the researchers. Then the researcher was the one who obtained consent from participants, and all participants were free to choose whether to participate or not. The in-charge did not ask participants anything about being approached by the researcher.

6 What was the employment relationship between the participants and the researchers? Given that the participants were employees in the hospital where the study was being conducted, was there a sense of obligation to participate (undue coercion?)? What was the power dynamic between the participants and the employer and/or the researcher? Was there a possibility that they might feel that their participation was necessary to ensure that their employment status was not jeopardised?

7 Authors’ Response

OThe relationship between the researchers and participants was limited strictly to the conduct of the study. All researchers were affiliated with Muhimbili University of Health and Allied Sciences, while participants were employees of Muhimbili National Hospital. Although both institutions share the name “Muhimbili,” they operate independently under distinct management structures, despite being located adjacent to one another.

8 Author contributions. Please clarify which of the authors conducted the interviews. The article indicates that a midwife was a co-facilitator, but it is not clear who was the lead interviewer. It is not clear if the midwife co-facilitator (information gatherer/data collector) is included in the authorship or acknowledgements. Please clarify this contribution.

9 Authors’ Response

Thank you for the comment. We have updated the author's contribution. The RA has also been included as a co-author because he participated in curating data and creating the initial draft of the manuscript, which warrants authorship.

Attachment Submitted filename: Response to Reviewewers.docx

# Editor report: Decision Letter 1

**DOI:** 10.1371/journal.pone.0354738.r004

**Authors:** Hannah Tappis

12 Jul 2026

Understanding the Underuse: Barriers to Vacuum-Assisted Birth Among Clinicians in Tanzania: An Exploratory Qualitative Study

PONE-D-25-41107R1

Dear Dr. Chikwala,

We’re pleased to inform you that your manuscript has been judged scientifically suitable for publication and will be formally accepted for publication once it meets all outstanding technical requirements.

Within one week, you’ll receive an e-mail detailing the required amendments. When these have been addressed, you’ll receive a formal acceptance letter and your manuscript will be scheduled for publication.

An invoice will be generated when your article is formally accepted. Please note, if your institution has a publishing partnership with PLOS and your article meets the relevant criteria, all or part of your publication costs will be covered. Please make sure your user information is up-to-date by logging into Editorial Manager at Editorial Manager® and clicking the ‘Update My Information' link at the top of the page. For questions related to billing, please contact billing support.

If your institution or institutions have a press office, please notify them about your upcoming paper to help maximize its impact. If they’ll be preparing press materials, please inform our press team as soon as possible -- no later than 48 hours after receiving the formal acceptance. Your manuscript will remain under strict press embargo until 2 pm Eastern Time on the date of publication. For more information, please contact onepress@plos.org.

Kind regards,

Hannah Tappis, DrPH, MPH

Academic Editor

PLOS One

Additional Editor Comments (optional):

Reviewers' comments:

# Editor report: Acceptance letter

**DOI:** 10.1371/journal.pone.0354738.r005

**Authors:** Hannah Tappis

PONE-D-25-41107R1

PLOS One

Dear Dr. Chikwala,

I'm pleased to inform you that your manuscript has been deemed suitable for publication in PLOS One. Congratulations! Your manuscript is now being handed over to our production team.

At this stage, our production department will prepare your paper for publication. This includes ensuring the following:

* All references, tables, and figures are properly cited

* All relevant supporting information is included in the manuscript submission,

* There are no issues that prevent the paper from being properly typeset

You will receive further instructions from the production team, including instructions on how to review your proof when it is ready. Please keep in mind that we are working through a large volume of accepted articles, so please give us a few days to review your paper and let you know the next and final steps.

Lastly, if your institution or institutions have a press office, please let them know about your upcoming paper now to help maximize its impact. If they'll be preparing press materials, please inform our press team within the next 48 hours. Your manuscript will remain under strict press embargo until 2 pm Eastern Time on the date of publication. For more information, please contact onepress@plos.org.

You will receive an invoice from PLOS for your publication fee after your manuscript has reached the completed accept phase. If you receive an email requesting payment before acceptance or for any other service, this may be a phishing scheme. Learn how to identify phishing emails and protect your accounts at https://explore.plos.org/phishing.

If we can help with anything else, please email us at customercare@plos.org.

Thank you for submitting your work to PLOS ONE and supporting open access.

Kind regards,

PLOS ONE Editorial Office Staff

on behalf of

Dr. Hannah Tappis

Academic Editor

PLOS One
